# Genome-Wide Identification and Expression Analysis of HD-ZIP I Gene Subfamily in *Nicotiana tabacum*

**DOI:** 10.3390/genes10080575

**Published:** 2019-07-30

**Authors:** Yueyue Li, Bingchuan Bai, Feng Wen, Min Zhao, Qingyou Xia, Da-Hai Yang, Genhong Wang

**Affiliations:** 1 State Key Laboratory of Silkworm Genome Biology, Biological Science Research Center, Southwest University, Chongqing 400716, China; 2 Chongqing Institute of Tobacco Science, Chongqing 400716, China; 3 Biological Science Research Center, Southwest University, Chongqing 400716, China; 4 Chongqing Engineering and Technology Research Center for Novel Silk Materials, Southwest University, Chongqing 400716, China; 5 Chongqing Key Laboratory of Sericulture, Southwest University, Chongqing 400716, China; 6 Tobacco Breeding and Biotechnology Research Center, Yunnan Academy of Tobacco Agricultural Sciences, Key Laboratory of Tobacco Biotechnological Breeding, National Tobacco Genetic Engineering Research Center, Kunming 650021, China

**Keywords:** *HD-Zip* I, gene subfamily, *Nicotiana tabacum*, abiotic stress, expression analysis

## Abstract

The homeodomain-leucine zipper (HD-Zip) gene family, whose members play vital roles in plant growth and development, and participate in responding to various stresses, is an important class of transcription factors currently only found in plants. Although the HD-Zip gene family, especially the HD-Zip I subfamily, has been extensively studied in many plant species, the systematic report on HD-Zip I subfamily in cultivated tobacco (*Nicotiana tabacum*) is lacking. In this study, 39 HD-Zip I genes were systematically identified in *N. tabacum* (Nt). Interestingly, that 64.5% of the 31 genes with definite chromosome location information were found to originate from *N. tomentosoformis*, one of the two ancestral species of allotetraploid *N. tabacum*. Phylogenetic analysis divided the *NtHD-Zip* I subfamily into eight clades. Analysis of gene structures showed that *NtHD-Zip* I proteins contained conserved homeodomain and leucine-zipper domains. Three-dimensional structure analysis revealed that most *NtHD-Zip* I proteins in each clade, except for those in clade η, share a similar structure to their counterparts in *Arabidopsis*. Prediction of cis-regulatory elements showed that a number of elements responding to abscisic acid and different abiotic stresses, including low temperature, drought, and salinity, existed in the promoter region of *NtHD-Zip* I genes. The prediction of *Arabidopsis* ortholog-based protein–protein interaction network implied that *NtHD-Zip* I proteins have complex connections. The expression profile of these genes showed that different *NtHD-Zip* I genes were highly expressed in different tissues and could respond to abscisic acid and low-temperature treatments. Our study provides insights into the evolution and expression patterns of *NtHD-Zip* I genes in *N. tabacum* and will be useful for further functional characterization of *NtHD-Zip* I genes in the future.

## 1. Introduction

As regulatory proteins for the transcriptional activation or repression of target genes, transcription factors (TFs) are the main players in development and differentiation in eukaryotic organisms [1]. The TFs regulate gene expression by specifically interacting with cis-regulatory regions [2]. The homeodomain-leucine zipper (HD-Zip) gene family is a relatively pivotal class of TFs, present only in the plant kingdom [3,4]. This gene family has been identified in multiple plant species, such as soybean (*Glycine max*), tomato (*Solanum lycopersicum*), grape (*Vitis vinifera*), rice (*Oryza sativa*), maize (*Zea mays*), and wheat (*Triticum aestivum*) [5,6,7,8,9,10]. Based on their corresponding gene structure, including some specific cis-elements, conserved sequences, and biological function, they can be divided into four subfamilies: HD-Zip I to HD-Zip IV [4]. The number of members in each HD-Zip subfamily varies. All HD-Zip proteins from HD-Zip I, II, III, and IV subfamilies contain HD and leucine zipper (LZ) motifs [11]. The HD can specifically bind to DNA, and the LZ can act as a dimerization motif. Members of HD-Zip I contain these two basic motifs. Besides the HD and LZ in the HD-Zip family members, other motifs have been described and functionally characterized in HD-Zip II, III, and IV. For example, some extra motifs called CPSCE (consisting of five conserved amino acid sequences Cys, Pro, Ser, Cys, and Glu) and N-terminal conserved sequence have been shown in the HD-Zip II subfamily [12,13]. The START (star-related lipid transfer) motif, a steroidogenic acute regulatory protein related to lipid transfer, was found in HD-Zip III and IV subfamilies [14,15]. It was reported that HD-Zip I and II proteins can recognize a similar pseudopalindromic sequence CAAT(C/G)ATTG [4,12,13], whereas HD-Zip III and IV proteins recognize sequences GTAAT(G/C)ATTAC and TAAATG(C/T)A, respectively [11,14,15].

The HD-Zip I subfamily has been shown to play important roles in plant growth, development, de-etiolation, and response to abiotic and biotic stresses [16,17,18,19]. There are 17, 13, 14, and 22 HD-Zip I members in *Arabidopsis thaliana*, *Cucumis sativus*, *O. sativa*, and *S. lycopersicum*, respectively [3,6,8,20]. In *Arabidopsis*, the ectopic expression of one HD-Zip I member *ATHB12* resulted in larger leaves with enlarged cells, strongly suggesting its positive effect on leaf growth [21]; another HD-Zip I member *ATHB1* was involved in cell wall composition and elongation [22]. In *C. sativus*, HD-Zip I member *CsGL1* was identified to function in trichome formation [23]. Ariel et al. demonstrated that *Medicago truncatula* HD-Zip I TF *HB1* was required for the adaptive developmental response of lateral roots [24]. Overexpression of rice *HD-Zip* I genes, *Oshox12*, and *Oshox14*, led to reduced panicle length and a dwarf phenotype [25]. In addition to function in plant growth and development, HD-Zip I proteins were shown to play important roles in abiotic stress responses. For instance, *ATHB7* and *ATHB12*, which were strongly up-regulated by water-deficit and abscisic acid, function as positive regulators of PP2C in *Arabidopsis* [26]. *HaHB4* was involved in the regulation of tolerance to water deficit through ethylene-mediated senescence in sunflower [27]. Both rice HD-Zip I members *Oshox22* and *OsSLI1* function in abscisic acid (ABA)-mediated drought and salt tolerance [28,29]. Overexpression of *Zmhdz10* or *TaHDZipI-5* could increase plant tolerance to low temperature, drought, or salt stress [30,31]. Soybean *HD-Zip* I gene *Gshdz4* was significantly up-regulated by alkali stress, and further results showed that it enhanced bicarbonate tolerance and response to osmotic pressure [32]. It was also reported that over-expressing one chickpea *HD-Zip* I gene, *CaHDZ12*, resulted in improved tolerance to abiotic stress, and *CaHDZ12* expression was regulated by *CaWRKY70* [33]. The expression of *Nicotiana attenuata* HD-Zip I member *NaHD20* was also shown to be positively correlated with ABA accumulation in leaves during water deficit and the expression of some dehydration-responsive genes [34]. In addition, HD-ZIP I members have also been shown to play a defensive role against some biotic stresses. An *Arabidopsis* mutant with constitutively overexpressed *ATHB13* exhibited resistance to downy mildew (*Hyaloperonospora arabidopsidis*) and green peach aphid (*Myzus persicae*) [35]. A HD-ZIP I protein in pepper was characterized as having a positive role in regulating resistance to *Ralstonia solanacearum* infection [36].

Although *HD-ZIP* I genes have been widely studied in other plant species, there are no reports concerning *HD-ZIP* I in allotetraploid *N. tabacum*. In this study, a genome-wide identification of *HD-ZIP* I genes was performed in *N. tabacum* (Nt). The phylogenetic relationship, conserved domains, genome organization, and gene structure of *NtHD-ZIP* I members were investigated. Also, possible cis-acting elements in the promoter sequences of *NtHD-ZIP* I genes and three-dimensional modeling of *NtHD-ZIP* I proteins were analyzed. Furthermore, expression profiles of *NtHD-ZIP* I genes were investigated in different tissues and under low temperature or ABA treatment using quantitative real-time PCR (qRT-PCR). This study provided essential information concerning the *NtHD-Zip* I subfamily genes in *N. tabacum* and enhanced our understanding of *HD-Zip* I genes in plants.

## 2. Materials and Methods

### 2.1. Sequence Search and Identification of HD-Zip I Subfamily

Predicted tobacco coding sequences and protein sequences were downloaded from the Sol Genomics Network (http://solgenomics.net/organism/). Sequences of *Arabidopsis*, *S. lycopersicum*, *Camellia sinensis*, *O. sativa*, *Z. mays*, and *Manihot esculenta* were downloaded from PlantGDB (http://www.plantgdb.org/) and were named according to previous reports (Table S1). Prediction of subcellular localization was based on WoLf (https://www.genscript.com/wolf-psort.html). Each *NtHD-ZIP* I gene was mapped to its corresponding chromosome and ancestral species based on the reported tobacco genome [37,38,39].

### 2.2. Phylogenetic, Gene Structure, and Conserved Domain Analyses of HD-Zip Class I Subfamily of Tobacco

MEGA 7.0 was used to construct the phylogenetic tree of HD-Zip I family proteins in *S. lycopersicum*, *C. sinensis*, *O. sativa*, *Z. mays*, *M. esculenta*, and *Arabidopsis* using the neighbor-joining algorithm with 1000 bootstrap replications. Gene structure was obtained by online program Gene Structure Display Server (http://gsds.cbi.pku.edu.cn) using *NtHD-Zip* I subfamily cDNA and genomic sequences. The online site Multiple Em for Motif Elicitation (MEME, http://meme.nbcr.net/meme/cgibin/meme.cgi) was used to identify conserved domains of NtHD-Zip I proteins, with a total of 20 motifs investigated, then processed through an online website (http://www.omicshare.com/). Multiple sequence alignments were done together by ClustalW and Genedoc (https://www.psc.edu/biomed/genedoc/) [40].

### 2.3. Analysis of Promoter, Prediction of Three-Dimensional Modeling, and Interacting Networks of HD-Zip Class I Proteins between N. tabacum and Arabidopsis

About 2000 bp of genomic DNA sequence at upstream of the starting codon of *NtHD-Zip* I genes were selected for analysis of *cis*-elements in promoter through the online website Genomatix (http://www.genomatix.de/solutions/genomatix-software-suite.html). In this study, ABA-, low temperature-, drought stress-, and salinity stress-regulated elements were selected to analyze promoter cis-elements. Three-dimensional modeling of HD-Zip I proteins was performed by Phyre2 server (Protein Homology/analogY Recognition Engine, http://www.sbg.bio.ic.ac.uk/phyre2) [41]. The prediction of the interacting networks of proteins was constructed in STRING (https://string-db.org/?tdsourcetag=s_pctim_aiom).

### 2.4. Plant Material and Stress Treatment

The cultivated *N. tabacum* variety Honghuadajingyuan (HHDJY) was used to detect the expression of *NtHD-Zip* I subfamily genes in various tissues. Seeds were sterilized in 75% ethanol for 30 s, washed 1–2 times with sterile water, then sterilized in 10% sodium hypochlorite for 8–10 min, washed 4–5 times with sterile water and thereafter seeded on MS solid medium, and cultured at 25 °C. The seedlings were cultured in 16/8 h of light/dark photoperiod at 25 °C. Roots, stems, and leaves were collected from tobacco seedlings about 2 months after germination. Furthermore, flowers and seeds were collected from mature tobacco in soil. Roots, stems, leaves, flowers, and seeds of tobacco were temporarily stored in liquid nitrogen and then stored in a −80 °C refrigerator for the detection of tissue expression profiles.

For low-temperature stress, tobacco seedlings with four leaves (approximately 2 months after germination) grew under 16/8 h of light/dark photoperiod at 25 °C in soil, were placed in a 4 °C incubator for low-temperature treatment. Tobacco leaves at time points of 0, 1, 6, 12, and 24 h were sampled for RNA extraction and quantitative analysis. For ABA treatment, seedlings with four leaves (approximately 2 months after germination) grew in the same conditions as those of low-temperature stress, and the leaves of plants were sprayed with 200 μM ABA; leaves were collected at 0, 1, 6, 12, and 24 h after the ABA treatment. Three biological replicates were taken at each time point under 4 °C and ABA treatments. All 30 treated plants were used for sample harvesting and the following qPCR analysis. 

### 2.5. RNA Extraction and Quantitative RT-PCR

Total RNA in tobacco was extracted through Trizol reagent (Invitrogen). The cDNA was synthesized by extracting RNA using GoScriptTM Reverse Transcription System (Promega). Gene-specific primers were designed in online tools (PrimerQuest Tool, https://sg.idtdna.com/Primerquest/Home/Index). Primers of target and reference genes (*NtEF1α*) were used for quantitative RT-PCR (Table S2) using Takara’s SYBR Premix Ex Taq^TM^II.

The real-time PCR analyses were made by using a qTOWER2.2 real-time PCR system (Analytik Jena AG, Jena, Germany), and the procedure was as follows: denaturation at 95 °C for 3 min, followed by 40 cycles of denaturation at 95 °C for 10 s and annealing/extension at 60 °C for 1 min [42]. Each sample was run through three technical repeats, and quantitative results were analyzed using the 2^−ΔΔCt^ algorithm [43]. Pictures were drawn using GraphPad Prism 6. The heat map used for tissue expression profiling was made by an online website (http://www.omicshare.com/) based on row scale-transformed expression values.

### 2.6. Statistical Analysis

GraphPad Prism 6 was utilized for all statistical analyses. ****, ***, **, and * indicate significant differences compared to the control (0 h) at *p* < 0.0001, *p* < 0.001, *p* < 0.01, and *p* < 0.05, respectively.

## 3. Results

### 3.1. Identification of HD-Zip Class I Members in N. tabacum

To identify the *NtHD-Zip* I subfamily gene in *N. tabacum*, a BLASTP search was implemented against the tobacco genome database using HD-Zip I protein sequences from multiple plants, and conserved HD and LZ domains were further analyzed following BLASTP search. A total of 39 *NtHD-Zip* I candidate genes were identified in tobacco and named *NtHDZI1*–*NtHDZI39* (Table 1). The length of the deduced protein sequences of the NtHD-Zip I members ranged from 172 (*NtHDZI38* and *NtHDZI39*) to 361 (*NtHDZI3*) amino acids. Results of subcellular localization prediction indicated that NtHD-Zip I members were all located in the nucleus (Table 1).

There were 37 *NsylHD-Zip* I genes found in ancestral species *N. sylvestris*, and *39 NtomHD-Zip* I genes in *N. tomentosoformis* according to two reported ancestral genomic databases (*N. sylvestris* and *N. tomentosoformis*) (Table S3) [39]. However, the complete genomic sequences *NtHDZI1–6*, *8*, *10*, *13*, *15*, *18*, *20*, *24*, *25*, *31*, and *35–39* only appeared in chromosomes of the T-genome (*tomentosiformis*) of *N. tabacum*, and *NtHDZI12*, *14*, *21*, and *32* were exclusively in chromosomes of the S-genome (*sylvestris*) of *N. tabacum.* The genomic origins of *NtHDZI7*, *9*, *11*, *16*, *17*, *19*, *22*, *23*, *26–30*, *33*, and *34* could not be determined using the current genomic maps [37,38]. Approximately 80% of *NtHD-Zip* I genes in tobacco could be located on the corresponding chromosome. In detail, there were four *NtHD-Zip* I genes on each chromosome 2, 12, and 13. Chromosome 17 contained the maximal number of *NtHD-Zip* I genes, seven in total. Each of chromosomes 4, 20, 23, and 24 contained two *NtHD-Zip* I genes respectively. Each of chromosomes 3, 7, 9, and 14 contained only one *NtHDZ* I gene (Table 1).

### 3.2. Phylogenetic and Evolutionary Analysis of HD-Zip Class I Proteins in N. tabacum

**Figure 1:**
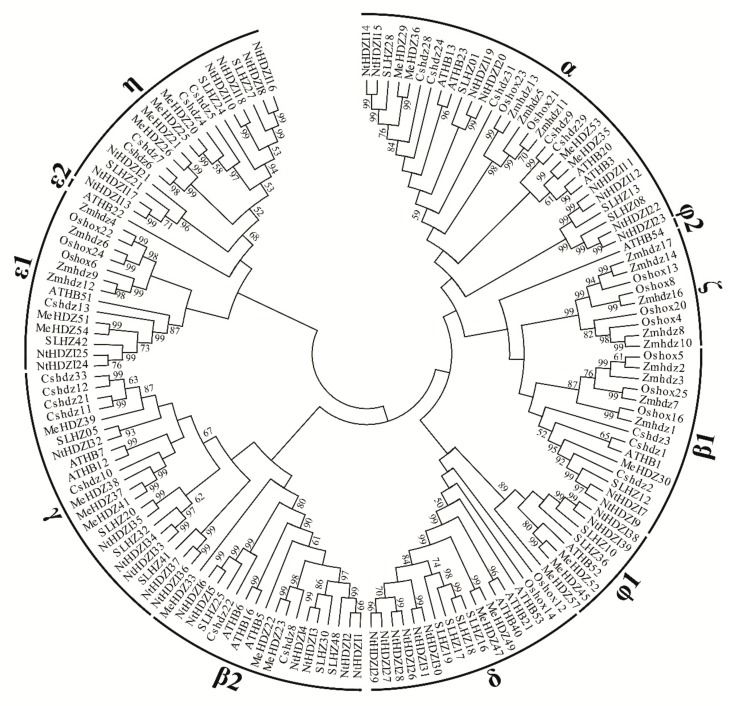
Phylogenetic analysis of HD-Zip I proteins from tobacco and other plants. The phylogenetic tree was constructed using 39, 17, 22, 14, 23, 16, and 20 HD-Zip I protein sequences from *N. tabacum*, *A. thaliana*, *S. lycopersicum*, *O. sativa*, *Cassava*, *Maize*, and *C. sinensis*, respectively. Plant HD-Zip I members were divided into eleven clades.

**Figure 2:**
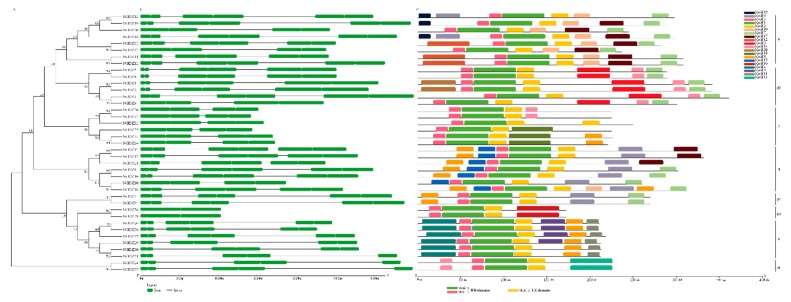
Gene structure, motif prediction of NtHD-Zip I proteins in *N. tabacum*. (**A**) The phylogenetic tree is constructed using the full-length amino acid sequences from *N. tabacum* alone by the NJ method. (**B**) Intron/exon structure of *NtHDZI* genes. Introns and exons are represented by black lines and green boxes, respectively. (**C**) Conserved motifs distribution of NtHDZI proteins. All motif predictions are obtained through the MEME online website (http://meme.nbcr.net/meme/cgibin/meme.cgi). Different color boxes at the top right-hand corner represent different motifs detected. The characteristic motifs, motif 1 and motif 3, corresponding to HD domain and motif 2 corresponding to LZ domain, are highlighted at the bottom.

A phylogenetic tree was constructed to assess the evolutionary relationships of NtHD-Zip I proteins among publicly available HD-Zip I proteins from other plants, including *A. thaliana*, *S. lycopersicum*, *O. sativa*, *Cassava*, *Maize,* and *C. sinensis* (Figure 1). Similar to previous results [3], there were 11 clades identified in the phylogenetic tree: α, β1, β2, γ, δ, ε1, ε2, η, φ1, φ2, and ζ. *NtHD-Zip* I members appeared in eight clades: α, β1, β2, γ, δ, ε1, η, and φ1. No NtHD-Zip Class I members were in clades ε2, φ2, and ζ. Additionally, NtHD-Zip Class I proteins were used alone to construct a phylogenetic tree, the classification results of which were consistent with that of the phylogenetic tree including HD-Zip Class I members from other plants (Figure 2A). Clade α contained the maximal number (eight) of NtHD-Zip Class I members: *NtHDZI11*, *12*, *14*, *15*, *19*, *20*, *22*, and 23. Each Clade β1, ε1, and φ1 had the fewest number (two) of NtHD-Zip I members.

In addition, similar to a phylogenetic tree for HD-Zip Class I members [20], clade β was divided into β1 and β2 sub-clades in the current analysis. Clade ε was also divided into sub-clades ε1 and ε2, and clade φ was divided into φ1 and φ2 (Figure 1). As found in the previous report, members of clade ζ belonged to monocots (rice and maize); members of clades β2, γ, ε2, η, φ1, and φ2 were only from dicots (*Arabidopsis*, tomato, *C. sinensis*, and *M. esculenta*) [20].

### 3.3. Genomic Structure, Conserved Domain, and Motif Analysis of HD-Zip Class I Proteins in N. tabacum

The exon–intron structure analysis showed that the *NtHD-Zip* Class I genes had either two or three exons, except for *NtHDZI38* and *NtHDZI39*, which had no intron (Figure 2B). Results of multiple sequence alignments showed that all NtHD-Zip I proteins contained conserved HD and LZ domains (Figure S1). Predicted motif (Figure 2C; Figure S2; Table S4) analysis revealed that motif 1 and motif 3 encoded the HD domain, and motif 2 encoded the LZ domain. Distribution features of the 20 predicted motifs in deduced proteins were consistent with the phylogenetic analysis (Figure 2).

### 3.4. Promoter Analysis of the HD-Zip Class I Genes in N. tabacum

To assess the possible response patterns of the *NtHD-Zip* I subfamily gene to different stress treatments, cis-acting elements including components related to stress treatment, such as the ABA-, low temperature-, drought-, and salinity-regulated elements were predicted in the promoter region of these genes (Table 2; Figure S4). The type and number of cis-components, which should respond to salinity, drought, and ABA in the promoter region of each gene, are listed in Table 2. The maximum number of such elements was found in *NtHDZI28* (107). Furthermore, the number of predicted cis-acting elements varied from 168 (*NtHDZI34*) to 237 (*NtHDZI28*) (Figure S3; Table S5). 

The promoter sequences of both *NtHDZI6* and *NtHDZI30* contained the fewest number of low temperature-regulated elements, with only 43 (Table S5). However, *NtHDZI22* contained the maximal number of low-temperature-regulated components with 69, followed by *NtHDZI21* with 67. There were more elements responding to ABA compared with those to low temperature. *NtHDZI28*, *13*, *38*, and *11* ranked in the top four for ABA-responding-elements, with 154, 155, 156, and 161, respectively. *NtHDZI23* contains the fewest number (107) of ABA-related components. 

*Arabidopsis* homeobox protein (AHBP), a classical cis-acting element, can respond to salinity, drought, and ABA [19,26,44,45,46], and was abundantly distributed in each of the NtHD-Zip Class I members. For example, there were 59 AHBP elements in *NtHDZI11*, which was the most compared to other *NtHD-Zip* I genes (Table S5). DNA binding with one finger (DOFF), responding to low-temperature stress [47], was another highly distributed element in NtHD-Zip Class I members—the promoter region of *NtHDZI3* contained the most number (24) of DOFF elements (Table S5). GT-box element (GTBX) is abundantly distributed among all the tested drought stress elements [48] and the promoter region of *NtHDZI12* has the maximum number of GTBX element. The numbers of cis-elements of *NtHDZI7* and *NtHDZI9* belonging to clade β1 were extremely similar (Figure 1), with 178 and 172, respectively. Likewise, the number of cis-elements in *NtHDZI38* and *NtHDZI39* of clade φ1 were 211 and 203, respectively (Figure 1; Table S5).

### 3.5. Prediction of Three-Dimensional Modeling and Interaction Network of HD-Zip Class I Proteins in N. tabacum and Arabidopsis

**Figure 3:**
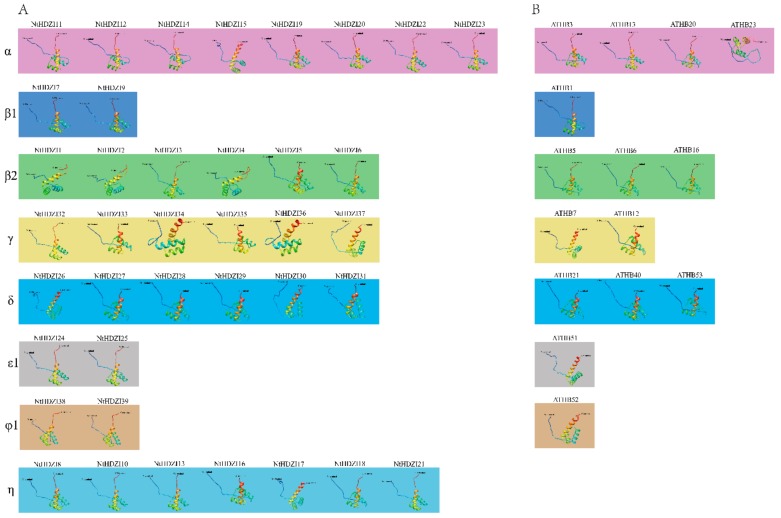
Three-dimensional modeling of HD-Zip I proteins in *N. tabacum* and *Arabidopsis.* The structure of NtHDZI proteins (**A**) and ATHBI proteins (**B**), with a confidence level > 90%, is shown and the activated sites are highlighted in light blue, yellow, and light green.

**Figure 4:**
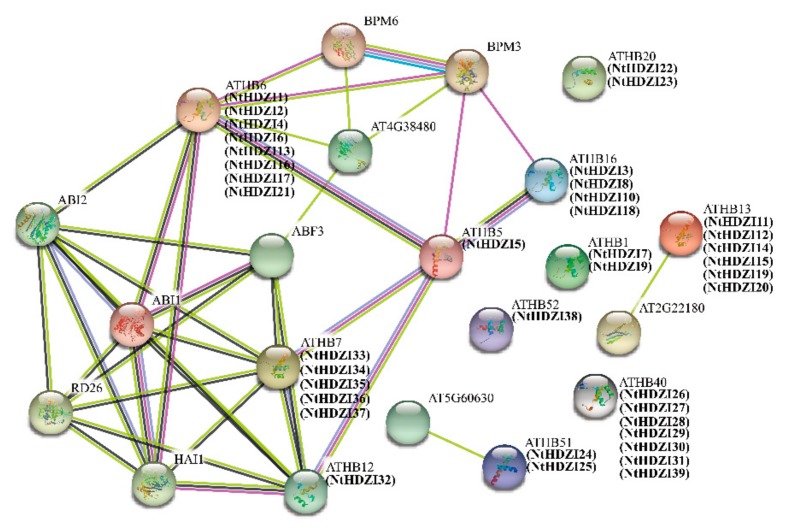
The prediction of the interaction network of NtHD-Zip I proteins based on the interactions of their orthologs in *Arabidopsis*.

To better understand the structural characteristics and interaction network of the HD-Zip I subfamily, three-dimensional models of all the NtHD-Zip I proteins were constructed using the Phyre2 server (Figure 3), and the protein–protein interactions of the HD-Zip I subfamily between *N. tabacum* and *Arabidopsis* were predicted using STRING (Figure 4). 

All NtHD-Zip I proteins contained the α-helix and coil structure [49]. Most NtHD-Zip I members shared a similar three-dimensional structure, especially for those clustered in the same clade. The predicted structures of NtHD-Zip proteins in each sub-clade were similar to their counterpart(s) in *Arabidopsis*, except for clade η members. 

The protein–protein interactions of HD-Zip I subfamily between *N. tabacum* and *Arabidopsis* were determined using STRING (http://string-db.org/). All NtHD-Zip Class I proteins appeared in the known interaction network of *Arabidopsis* HD-Zip Class I proteins according to the prediction, implying that they had complex connections. The results showed that the protein structure and sequence of ATHB7 were similar to five NtHD-Zip I proteins (NtHDZI33–37), and ATHB12 was similar to NtHDZI32, suggesting that these NtHD-Zip I members may be involved in both regulating growth or development, and responding to water deficit [44]. In addition, like their counterpart ATHB20, NtHDZI22 and 23 might play a role in regulating ABA sensitivity and seed dormancy [50]. There were six *NtHD-Zip* I genes (*NtHDZI11*, *12*, *14*, *15*, *19*, and *20*) similar to *ATHB13*, which may represent a component of the sucrose-signaling pathway [18]. ATHB16 was shown to be a regulator of flowering time in response to photoperiod [17]. According to the predicted interaction network, NtHDZI3, 8, 10, and 18 might play a similar role in *N. tabacum.* Similarly, NtHDZI24 and 25 might function as a LATE MERISTEM IDENTITY1 (LMI1) homeodomain protein to regulate stipular ratio similar to the role of ATHB51 [51], whereas NtHDZI1, 2, 4, 6, 13, 16, 17, and 21 might be involved in regulating the response to water-deficit in an ABA-dependent manner similar to the function of ATHB6 [45].

### 3.6. Expression Profiles of Tobacco HD-Zip I Genes in Various Tissues

**Figure 5:**
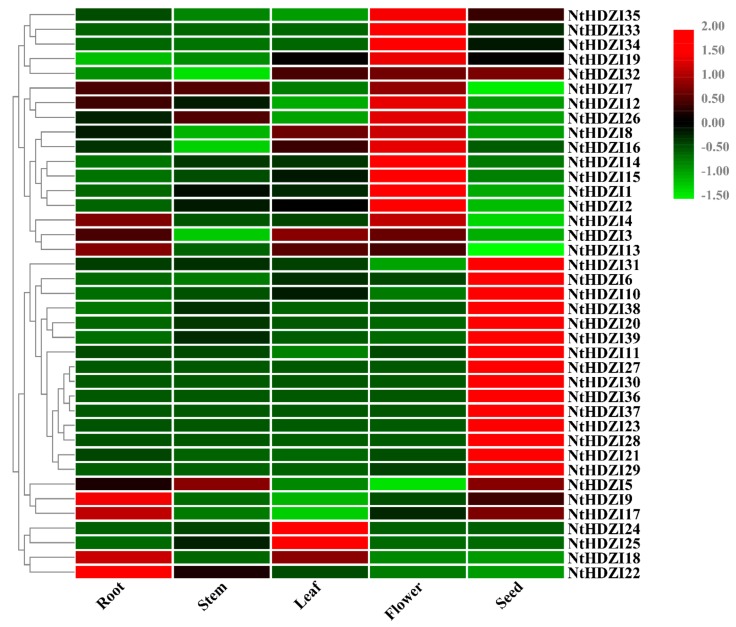
Relative expression levels of *NtHDZI* genes in various tissues. The roots, stems, leaves, flowers, and seeds were collected from the *N. tabacum* plants. All of the expression levels of the *NtHDZI* genes were normalized by the expression levels of *NtEF1α*. The 2^−ΔΔCT^ method was used to be an evaluation of the relative expression. The heat map was drawn in row scale-transformed expression values. Red or green colors represent the difference in expression levels in each sample, respectively.

To gain insight into potential functions, quantitative RT-PCR was employed to determine the expression patterns of *NtHD-Zip* I genes in five tissues: roots, stems, leaves, flowers, and seeds (Figure 5). All 39 *NtHD-Zip* I genes were expressed in one or more tested tissues with different expression patterns. 

*NtHDZI1*, *2*, *14–16*, *26*, and *33–35* had the highest expression in flowers compared with other tissues. Fifteen NtHD-Zip I genes: *NtHDZI6*, *10*, *11*, *20*, *21*, *23*, *27–31*, and *36–39* showed higher expression levels in seeds and some were even expressed in a seed-specific manner. Interestingly, *NtHDZI24* and *NtHDZI25* were exclusively expressed in leaves, and *NtHDZI22* was predominantly expressed in roots. *NtHDZI7* and *NtHDZI12* had higher expression in roots, stems, and flowers, and *NtHDZI19* and *NtHDZI32* were mainly expressed in leaves, flowers, and seeds. In addition, *NtHDZI5* had higher expression levels in roots, stems, and seeds, and *NtHDZI8* was preferentially expressed in roots, leaves, and flowers. *NtHDZI9* and *NtHDZI17* showed higher expression in roots and seeds, and *NtHDZI13* and *NtHDZI18* were highly expressed in roots and leaves. Compared to other genes, *NtHDZI4* showed relatively wider expression patterns in roots and flowers. Furthermore, *NtHDZI3* showed a relatively stable expression pattern among all tissues tested. Interestingly, members within each clade did not always share similar expression patterns.

### 3.7. Expression Profiling of Tobacco HD-Zip I Genes under Low Temperature and ABA Treatments

**Figure 6:**
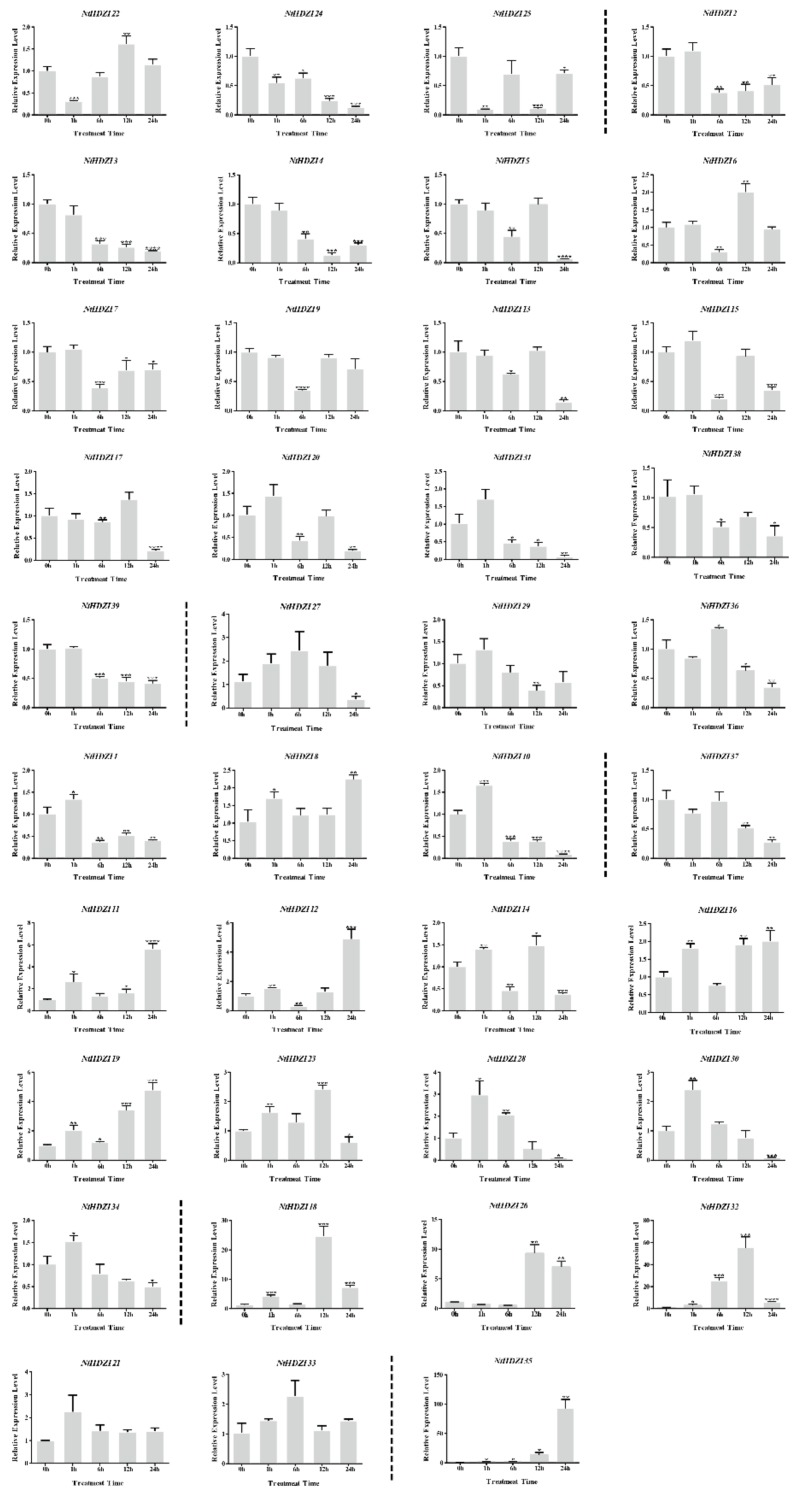
The expression levels of 39 *NtHD-Zip* I genes in *N. tabacum* under low-temperature (4 °C) treatment. Bars represent the mean values of three replicates ± standard deviation (SD). All of the expression levels of the *NtHDZI* genes were normalized by the expression levels of *NtEF1α*. Untreated leaves (0 h) were normalized as “1” at each graph.****, ***, **, and * indicate significant difference compared to the control (0 h) at *p* < 0.0001, *p* < 0.001, *p* < 0.01, and *p* < 0.05, respectively.

**Figure 7:**
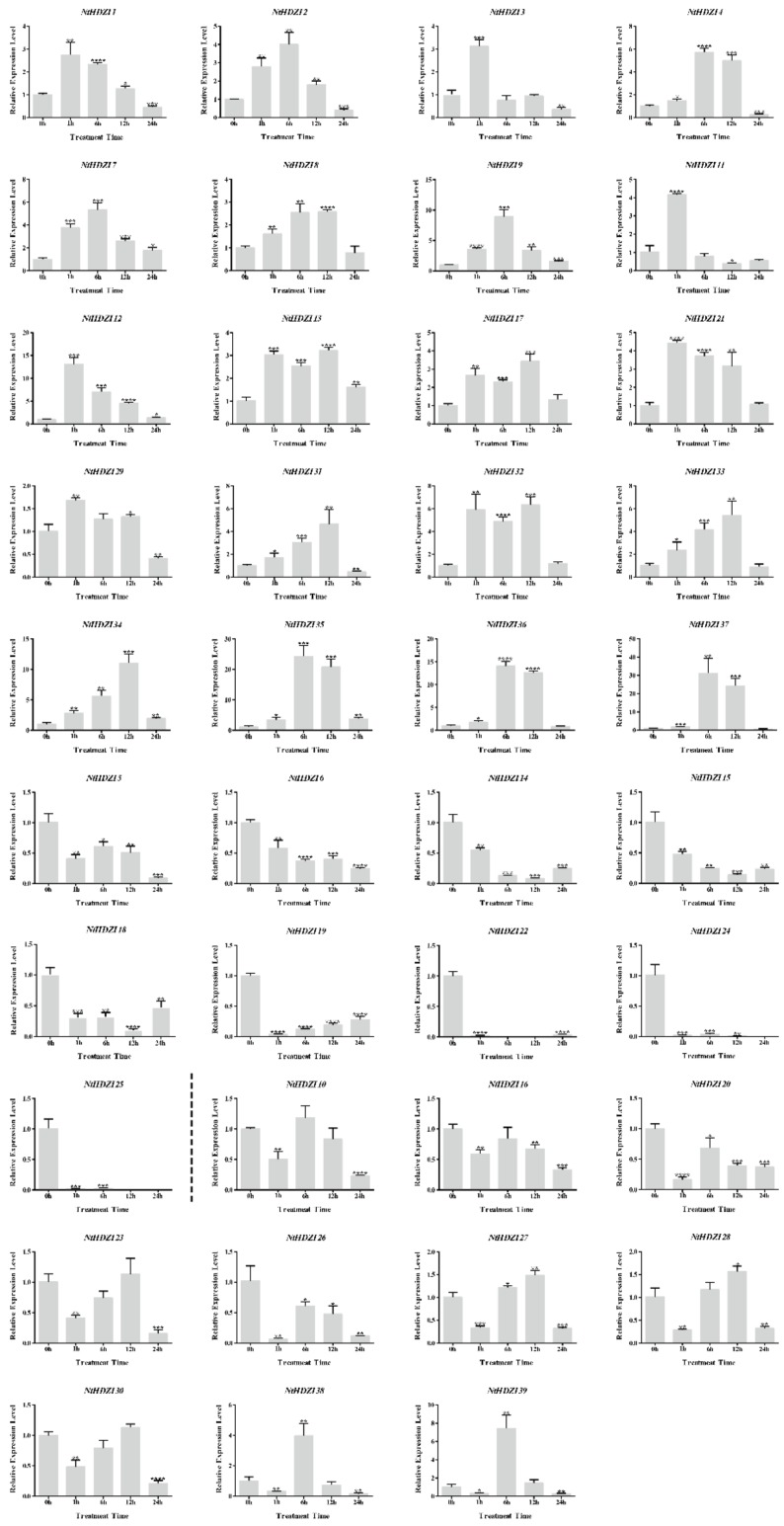
The expression levels of 39 *NtHD-Zip* I genes in *N. tabacum* under ABA (200 μM) treatment. Bars represent the mean values of three replicates±standard deviation (SD). All of the expression levels of the *NtHDZI* genes were normalized by the expression levels of *NtEF1α*. Untreated leaves (0 h) were normalized as “1” at each graph.****, ***, **, and * indicate significant difference compared to the control (0 h) at *p* < 0.0001, *p* < 0.001, *p* < 0.01, and *p* < 0.05, respectively.

The HD-Zip I genes have been reported to play important roles in response to abiotic stress. Transcript abundances of *NtHD-Zip* I genes were investigated in leaves under low temperature (Figure 6) and ABA treatments (Figure 7). The results suggested that most *NtHD-Zip* I genes were responsive to low-temperature stress and ABA treatment, with the exception of *NtHDZI21* and *NtHDZI33.* Expression of *NtHDZI22*, *24*, and *25* exhibited rapid down-regulation at 1 h after exposure to low temperature, but *NtHDZI2–7*, *9*, *13*, *15*, *17*, *20*, *31*, *38*, and *39* were significantly down-regulated at 6 h after exposure to low temperature (Figure 6). Expression of some genes, for example, *NtHDZI27*, 29, 36, and 37, declined at a late stage after plant exposure to low temperature. With low-temperature treatment, *NtHDZI1*, *8*, *10–12*, *14*, *16*, *19*, *23*, *28*, *30*, and *34* were up-regulated at 1 h, whereas expression of *NtHDZI18*, *26*, *32*, and *35* increased greatly and significantly at 12 or 24 h. Interestingly, *NtHDZI21* and *NtHDZI33* expression did not significantly change at all time points tested after low-temperature treatment.

The expression patterns of *NtHD-Zip* I genes were roughly divided into three types under ABA treatment (Figure 7). The first type was characterized by an expression pattern with significant up-regulation at 1 h; these genes were *NtHDZI1–4*, *7–9*, *11–13*, *17*, *21*, *29*, and *31–37.* The second type of expression pattern was continuous down-regulation under ABA treatment, including *NtHDZI5*, *6*, *14*, *15*, *18*, *19*, *22*, *24*, and *25*. The third type of expression patterns was a transient decline at 1 h, recovered or even enhanced at 6 or 12 h, and then declined again at 24 h. Genes with the third type of expression pattern were *NtHDZI10*, *16*, *20*, *23*, *26–28*, *30*, *38*, and *39.*

## 4. Discussion

Plants are confronted with major challenges involving biotic and abiotic stresses. The TFs, as regulatory proteins regulating gene expression by binding to cis-acting elements on the promoter [2], are responsive to various stresses. The HD-Zip proteins are a type of TF found only in plants, and play important roles in plant growth and stress response [3,4]. The HD-Zip family consists of four subfamilies based on the reported identification in other species: HD-Zip I–IV [11]. The HD-Zip I subfamily is mainly involved in stress response, such as to low temperature, drought, salt, and ABA [5,6,19]. To date, although HD-Zip I members have been extensively studied in other species [6,20,52,53,54], no detailed identification has been performed in the model plant tobacco. In this study, 39 *NtHD-Zip* I genes were identified in *N. tabacum* by comprehensive analysis. Tobacco has more HD-Zip I genes (39) compared with other plants—17, 22, 20, 14, and 16 in *Arabidopsis*, tomato, tea plant, rice, and maize, respectively [3,6,8,9,55]. 

As an allotetraploid plant, cultivated tobacco was evolved through interspecific hybridization of *N. sylvestris* and *N. tomentosiformis*, and the origin of these HD-Zip I genes were surveyed for chromosome localization [37,38]. The complete genomic sequences of *NtHDZI1–6*, *8*, *10*, *13*, *15*, *18–20*, *24*, *25*, *31*, and *35–39* were only found in the T-genome, while those of *NtHDZI12*, *14*, *21*, and *32* only appeared in the S-genome of *N. tabacum* (Table 1). The uneven distribution of these *NtHD-Zip* I genes implied that complex gene rearrangement and deletion occurred following interspecific hybridization. 

Importantly, similarly to HD-Zip I in other plant species [6,7,8,20], all identified NtHD-Zip I proteins contained conserved HD and LZ domains (Figure 2 and Figure S1). There were 2–3 exons for most *NtHD-Zip* I genes, except for *NtHDZI38* and *NtHDZI39*, which had just one exon. Phylogenetic analysis showed that *NtHD-Zip* I genes were distributed in 8 out of 11 plant HD-Zip I subclasses, indicating that the basic features of the plant HD-Zip I gene family were formed early, which was also independently confirmed by the intron–exon structure and motif organization patterns (Figure 1, Figure 2, and Figure S2; Table S4).

There were 14 genes in pairs in the phylogenetic tree: *NtHDZI1* and *NtHDZI2*, *NtHDZI8* and *NtHDZI16*, *NtHDZI13* and *NtHDZI17*, *NtHDZI24* and *NtHDZI25*, *NtHDZI33* and *NtHDZI34*, *NtHDZI36* and *NtHDZI37*, and *NtHDZI38* and *NtHDZI39* (Figure 2). In addition to high sequence similarities, similar genomic structure, motif composition, and three-dimensional structure (Figure 2; Figure S2; Table S4), most pairwise genes: *NtHDZI1* and *NtHDZI2*, *NtHDZI13* and *NtHDZI17*, *NtHDZI24* and *NtHDZI25*, *NtHDZI33* and *NtHDZI34*, *NtHDZI36* and *NtHDZI37*, and *NtHDZI38* and *NtHDZI39*, tended to have a similar expression pattern in response to low temperature stress or ABA treatment (Figure 6 and Figure 7). For instance, both *NtHDZI36* and *NtHDZI37* were induced to the highest expression level at 6 h post-ABA stimuli, whereas *NtHDZI38* and *NtHDZI39* showed a dramatic reduction after 1 h for under low-temperature treatment and were significantly induced at 6 h by ABA treatment (Figure 7). In addition, *NtHDZI1* and *NtHDZI2* both showed a sharp reduction at 6 h under low-temperature stress (Figure 6). Interestingly, most of these paired HD-Zip I genes originated from *N. tomentosiformis* and derived from the same chromosome, for example, *NtHDZI1* and *NtHDZI2* in chromosome 13, *NtHDZI36* and *NtHDZI37* in chromosome 23, and *NtHDZI38* and *NtHDZI39* in chromosome 2 (Table 1). This implied that gene rearrangement or duplication occurred after interspecific hybridization. One exception was *NtHDZI13* and *NtHDZI17*, which exhibited similar expression patterns under low-temperature stress or ABA treatment. However, there were also paired genes which showed different expression patterns in response to low-temperature stress or ABA treatment, such as *NtHDZI8* and *NtHDZI16*–*NtHDZI8* were up-regulated, but *NtHDZI16* was down-regulated at 1 h under ABA treatment (Figure 7). Tomato *SLHZ08*, *SLHZ13*, and *Arabidopsis ATHB13* all belong to clade α, and expression of *SLHZ08*, *SLHZ13*, and *ATHB13* are up-regulated by low-temperature stress [6,56]. Expression of *SLHZ08* and *SLHZ13* can be up-regulated 3 h after the cold treatment [6]. Moreover, the expression level of *AtHB13* could also be induced, and overexpression of *AtHB13* conferred cold tolerance in *Arabidopsis* [56]. *NtHDZI11*, *12*, *14*, *19*, *22* and *23* were NtHD-Zip I family members in clade α (Figure 1), and expression of these genes were all up-regulated by low-temperature stress. Furthermore, most genes in this clade had a similar motif composition (Figure 2). Thus, these *NtHD-Zip* I genes might be involved in low-temperature tolerance.

Analysis of three-dimensional structure and protein–protein interactions are helpful to gain insights into the function of NtHD-Zip I members. Results of three-dimensional modeling indicated that most genes in the same clades tended to have similar protein structures (Figure 3). For instance, NtHDZI20 and NtHDZI22 of clade α showed similar protein models; and NtHDZI28 and NtHDZI29 of clade δ also had similar protein structures. Furthermore, a predicted interaction network of HD-Zip I proteins between tobacco and *Arabidopsis* was constructed (Figure 4). Previous reports showed that AtHB7 and AtHB12 functioned as positive transcriptional regulators of *PP2C* genes that participate in negative regulation of ABA signals [26]. Therefore, the tobacco counterparts *NtHDZI32–37* might share similar functions to those of *AtHB7* and *AtHB12*, which was further supported by the evolutionary analysis. Phylogenetic analysis revealed that *NtHDZI32–37* belongs to the same clades γ with *AtHB7* and *AtHB12* (Figure 1). Furthermore, expression profiling indicated the significant induction of these six genes (*NtHDZI32–37*) after ABA treatment, which suggests that *NtHDZI32–37* might participate in ABA-mediated signaling pathways. In addition, *NtHDZI11*, *12*, *14*, *15*, *19*, and *20* may play an active role in the sucrose-signaling pathway as *ATHB13* does [18], whereas *NtHDZI3*, *8*, *10*, and *18* may participate in regulating the photoperiod process as does *ATHB16* does [17].

The expression profile analysis showed that *NtHD-Zip* I genes had large expression variations among different tissues, which implied that they might have diverse functions. *NtHDZI1*, *2*, *14–16*, *26*, and *33–35* had higher expression levels in flowers (Figure 5). It was previously reported that *GhHB12*, specifically expressed in axillary buds, could affect cotton morphological construction and flower development delay when overexpressed [57]. *NtHDZI6*, *10*, *11*, *20*, *21*, *23*, *27–31*, and *36–39* showed a seed-specific expression pattern (Figure 5), implying a role in seed formation and seed dormancy similar to *ATHB20*, which was also abundantly expressed in seed germination of *Arabidopsis* [50]. The ectopic expression of *HaHB4*, an HD-Zip I TF from *Helianthus annuus*, could significantly change the morphology of the veins [58]. Given that NtHDZI24 and NtHDZI25 showed a relatively high expression in leaves, *NtHDZI24* and *NtHDZI25* might associate with tobacco leaf development. 

Many studies have reported that the HD-Zip I subfamily participates in abiotic stress response [6,19,26,27,28,29,30,31,32,33,45,50,56,59,60,61]. Cabello et al. found that *HaHB1* or *AtHB13* can be up-regulated by drought and salt and can significantly improve salt tolerance and drought resistance by their ectopic expression to maintain cell membrane integrity [59]. Furthermore, silencing of *SlHB2* can significantly increase the water content of plants and malondialdehyde contents to enhance the tolerance of plants to salt and drought in tomato [60]. Overexpressing an HD-Zip I gene from wheat endosperm, *TaHDZipI-2*, improves the freezing tolerance of plants [61]. In this study, we investigated the potentially abiotic stress-responding elements, including ABA, low temperature, drought stress, and salinity stress-regulated elements, within 2000-bp upstream of the start codon ATG (Table 2; Figure S4). Results showed that all the *NtHD-Zip* I genes contained a number of these stress-responding elements. The ABA-responding component accounted for the greatest number in the promoter region of *NtHD-Zip* I genes, elements related to drought and salinity stresses ranked in second place, and were followed by low-temperature stress components. A quantitative RT-PCR based expression profile revealed that expression levels of all *NtHD-Zip* I genes, except for *NtHDZI21* and *NtHDZI33*, showed significant changes, either up-regulated or down-regulated under low temperature or ABA treatment (Figure 6 and Figure 7), which is consistent with the results from the analysis of stress-regulated elements within the promoter sequence of *NtHD-Zip* I genes.

Although the potential functions were suggested based on the comparing of *NtHDZI* genes and deduced NtHDZI proteins with the members in other plant HD-Zip I subfamily (particularly with those in *Arabidopsis*) for exon–intron structure, predicted cis-acting elements associated with abiotic stress factors in the promoter regions, expression profiles, and homology, three-dimensional structures of proteins, putative protein–protein networks, respectively, the further functional characterizations of *NtHDZI* genes are required for understanding the biological significance of HD-Zip I subfamily in *N. tabacum*.

## 5. Conclusions

In total, 39 *NtHD-Zip* I genes were identified in this study. The *NtHD-ZIP* I gene subfamily were grouped into eight clades based on the evolutionary analysis. Gene structure, conserved motifs, and three-dimensional modeling were further analyzed. Genes grouped into the same clade tended to have similar exon-intron organization, motifs, and protein structure. Moreover, the upstream sequences of *NtHD-Zip* I genes contained abundant cis-acting elements associated with ABA response and abiotic stresses, such as low temperature, drought, or salinity response elements. The protein–protein interaction network was shown for tobacco and *Arabidopsis* HD-Zip I proteins. There were different expression patterns among different tissues, under ABA treatment, and low-temperature stress. This study will provide useful information for the functional characterizing *HD-Zip* I genes in *N. tabacum*.

## Figures and Tables

**Table 1:** Basic characteristics of tobacco homeodomain-leucine zipper (*HD-ZIP)* I genes.

Gene Name	Length of Deduced Protein (aa)	Number of Exons	Subcellular Location	Chromosome	Origin
*NtHDZI1*	342	3	nucl: 13.5, cyto_nucl: 7.5	Chr13	T
*NtHDZI2*	342	3	nucl: 13.5, cyto_nucl: 7.5	Chr13	T
*NtHDZI3*	361	3	nucl: 14	Chr13	T
*NtHDZI4*	301	2	nucl: 14	Chr13	T
*NtHDZI5*	289	3	nucl: 14	Chr12	T
*NtHDZI6*	290	3	nucl: 14	Chr12	T
*NtHDZI7*	270	3	nucl: 14	unknown	unknown
*NtHDZI8*	302	3	nucl: 13.5, cyto_nucl: 7.5	Chr24	T
*NtHDZI9*	270	3	nucl: 14	unknown	unknown
*NtHDZI10*	260	2	nucl: 14	Chr12	T
*NtHDZI11*	308	3	nucl: 13.5, cyto_nucl: 7.5	Chr17	unknown
*NtHDZI12*	308	3	nucl: 13.5, cyto_nucl: 7.5	Chr3	S
*NtHDZI13*	328	3	nucl: 14	Chr4	T
*NtHDZI14*	300	3	nucl: 14	Chr20	S
*NtHDZI15*	298	3	nucl: 14	Chr24	T
*NtHDZI16*	289	3	nucl: 14	unknown	unknown
*NtHDZI17*	332	3	nucl: 14	unknown	unknown
*NtHDZI18*	312	3	nucl: 14	Chr12	T
*NtHDZI19*	289	3	nucl: 14	unknown	unknown
*NtHDZI20*	245	2	nucl: 14	Chr9	T
*NtHDZI21*	288	3	nucl: 14	Chr20	S
*NtHDZI22*	282	3	nucl: 13.5, cyto_nucl: 7.5	Chr17	unknown
*NtHDZI23*	237	2	nucl: 14	Chr17	unknown
*NtHDZI24*	226	3	nucl: 14	Chr2	T
*NtHDZI25*	226	3	nucl: 14	Chr2	T
*NtHDZI26*	210	3	nucl: 14	Chr17	unknown
*NtHDZI27*	218	3	nucl: 14	Chr17	unknown
*NtHDZI28*	210	3	nucl: 14	Chr17	unknown
*NtHDZI29*	212	3	nucl: 14	Chr17	unknown
*NtHDZI30*	213	3	nucl: 14	unknown	unknown
*NtHDZI31*	212	3	nucl: 14	Chr14	T
*NtHDZI32*	250	2	nucl: 14	Chr7	S
*NtHDZI33*	226	2	nucl: 14	unknown	unknown
*NtHDZI34*	221	2	nucl: 14	unknown	unknown
*NtHDZI35*	225	2	nucl: 14	Chr4	T
*NtHDZI36*	225	2	nucl: 14	Chr23	T
*NtHDZI37*	225	2	nucl: 14	Chr23	T
*NtHDZI38*	172	1	nucl: 14	Chr2	T
*NtHDZI39*	172	1	nucl: 14	Chr2	T

“T“ and “S” represent the two origins of tobacco, *N. Tomentosoformis* and *N. sylvestris*, respectively. “Unknown” represented no judgment could be made on the origins of *NtHDZI* genes based on the available genome sequences.

**Table 2:** The number of stress-responsive cis-acting elements in the promoter region of each *NtHDZI* gene.

Gene Name	Respond to ABA (AREF, GLKF, LEGB, TCPF, SWNS)	Respond to Low Temperature (CGCG, DOFF, MADS, OCSE)	Respond to Salinity (ASRC, NIGS, NTMF)	Respond to Drought (CAAT, GARP, GTBX, PNRE)	Respond to Salinity, Drought, and ABA (ABRE, AHBP, MIIG, MYBL, MYBS, NACF)	Respond to Low Temperature, Salinity, and Drought (CCAF, MYCL)	Respond to Low Temperature, Salinity, and Drought (CE1F)	Respond to Low Temperature, Salinity, Drought, and ABA(DREB, EPFF, GCCF, WBXF)	Respond to Salinity and ABA (GBOX, JARE, TELO)
*NtHDZI1*	12	34	14	29	95	18	1	7	3
*NtHDZI2*	12	35	11	30	96	13	1	9	1
*NtHDZI3*	9	34	9	31	65	18	1	13	5
*NtHDZI4*	19	26	16	33	78	20	0	12	5
*NtHDZI5*	16	34	8	27	73	21	1	6	6
*NtHDZI6*	8	31	13	31	88	5	0	7	4
*NtHDZI7*	14	19	13	22	80	9	3	14	4
*NtHDZI8*	21	20	16	31	96	15	0	15	6
*NtHDZI9*	12	25	11	23	66	14	2	12	7
*NtHDZI10*	12	29	15	22	82	15	0	14	6
*NtHDZI11*	11	20	14	27	106	21	0	16	7
*NtHDZI12*	7	26	10	38	95	19	0	9	3
*NtHDZI13*	19	24	11	19	86	22	0	13	10
*NtHDZI14*	17	33	15	19	75	24	0	7	8
*NtHDZI15*	20	29	11	19	73	21	0	6	12
*NtHDZI16*	17	24	13	28	95	17	0	6	1
*NtHDZI17*	16	20	11	28	72	29	1	8	5
*NtHDZI18*	9	24	16	24	77	23	0	17	17
*NtHDZI19*	15	25	11	18	74	17	0	4	8
*NtHDZI20*	11	31	16	18	92	21	3	9	8
*NtHDZI21*	18	37	10	29	75	21	0	9	7
*NtHDZI22*	17	37	17	29	78	23	1	9	4
*NtHDZI23*	9	29	16	32	64	19	1	11	4
*NtHDZI24*	11	25	17	34	98	13	0	13	4
*NtHDZI25*	13	32	20	27	82	11	1	9	6
*NtHDZI26*	12	27	17	31	79	7	1	9	7
*NtHDZI27*	19	23	14	35	103	13	0	10	6
*NtHDZI28*	10	30	14	39	107	21	0	11	5
*NtHDZI29*	17	29	10	24	86	10	0	11	8
*NtHDZI30*	13	19	11	23	81	15	0	9	5
*NtHDZI31*	6	20	16	28	93	14	0	14	9
*NtHDZI32*	18	29	13	25	92	11	0	8	7
*NtHDZI33*	12	12	7	20	77	23	0	13	9
*NtHDZI34*	6	24	13	20	69	18	1	13	4
*NtHDZI35*	15	25	17	25	89	20	2	6	10
*NtHDZI36*	16	32	10	21	82	20	0	11	7
*NtHDZI37*	28	29	8	27	83	18	0	7	14
*NtHDZI38*	23	26	5	24	96	15	0	12	10
*NtHDZI39*	18	29	11	29	86	14	0	7	9

The numbers in the table represent the number of cis-acting elements. ABRE: ABA response elements; AHBP: Arabidopsis homeobox protein; AREF: Auxin response element; CCAF: Circadian control factors; DREB: Dehydration responsive element binding factors; EPFF: EPF-type zinc finger factors; GBOX: Plant G-box/C-box bZIP proteins; GCCF:GCC box family; GLKF: Golden2-like factors; JARE: Jasmonate response element; LEGB: Legumin Box family; MIIG: MYB IIG-type binding sites; MYBL:MYB-like proteins; MYCL: Myc-like basic helix-loop-helix binding factors; NACF: Plant specific NAC; SWNS: Secondary wall NACs; TCPF: DNA-binding proteins with the plant specific TCP-domain; TELO: Telo box (plant interstitial telomere motifs); WBXF: W Box family; CGCG:Calmodulin binding/CGCG box binding proteins; DOFF: DNA binding with one finger (DOF); CE1F:Coupling element 1 binding factors; MADS: MADS box proteins; OCSE: Enhancer element first identified in the promoter of the octopine synthase gene (OCS) of the Agrobacterium tumefaciens T-DNA; ASRC: AS1/AS2 repressor complex; MYBS: MYB proteins with single DNA binding repeat; NIGS: NACL-inducible genes; NTMF: NAC factors with transmembrane motif; CAAT: CAAT binding factors; GARP: MYB-related DNA binding proteins(Golden2, ARR, Psr); GTBX: GT-box elements.

## Supplementary Materials

The following are available online at https://www.mdpi.com/2073-4425/10/8/575/s1, Figure S1: Multiple sequence alignments in NtHD-Zip I proteins, Figure S2: Sequence logos of NtHDZI motifs in N. tabacum. The overall height of the stack represents the level of sequence conservation. The height of the residue in the stack represents the relative frequency of each residue at that location, Figure S3: The distribution of different stress-related cis-elements predicted in the promoter region of NtHD-Zip I genes, Figure S4: Promoter Analysis of HD-ZIP I genes in tobacco. A sequence of 2000 bp upstream of the start codon ATG of HD-Zip I genes in N. tabacum were investigated to analyze cis-elements by using Genomatix. Putative cis-elements were represented by different colors and distributed on the black lines, Table S1: Protein sequences of HD-Zip I in Nicotiana tabacum, Camellia sinensis, Solanum lycopersicum, Oryza sativa, Manihot esculenta, Zea mays, and Arabidopsis thaliana, Table S2: Primer sequences used for the qPCR analysis of HD-Zip I gene expression in tobacco, Table S3: Distribution of NtHD-ZIP I gene in ancestral species N. sylvestris and N. tomentosiformis, Table S4: Predicted motif sequence in NtHD-Zip I proteins, Table S5: Classification and statistics of different kinds of cis-acting elements in the promoter regions of NtHDZI genes.
